# Avoidant Responses to Interpersonal Provocation Are Associated with Increased Amygdala and Decreased Mentalizing Network Activity

**DOI:** 10.1523/ENEURO.0337-16.2017

**Published:** 2017-06-27

**Authors:** Macià Buades-Rotger, Frederike Beyer, Ulrike M. Krämer

**Affiliations:** 1 Department of Neurology, University of Lübeck, Lübeck 23562, Germany; 2 Institute of Psychology II, University of Lübeck, Lübeck 23562, Germany; 3 Institute of Cognitive Neuroscience, University College London, London WC1N 3AR, United Kingdom

## Abstract

When intentionally pushed or insulted, one can either flee from the provoker or retaliate. The implementation of such fight-or-flight decisions is a central aspect in the genesis and evolution of aggression episodes, yet it is usually investigated only indirectly or in nonsocial situations. In the present fMRI study, we aimed to distinguish brain regions associated with aggressive and avoidant responses to interpersonal provocation in humans. Participants (thirty-six healthy young women) could either avoid or face a highly (HP) and a lowly (LP) provoking opponent in a competitive reaction time task: the fight-or-escape (FOE) paradigm. Subjects avoided the HP more often, but retaliated when facing her. Moreover, they chose to fight the HP more quickly, and showed increased heart rate (HR) right before confronting her. Orbitofrontal cortex (OFC) and sensorimotor cortex were more active when participants decided to fight, whereas the mentalizing network was engaged when deciding to avoid. Importantly, avoiding the HP relative to the LP was associated with both higher activation in the right basolateral amygdala and lower relative activity in several mentalizing regions [e.g., medial and inferior frontal gyrus (IFG), temporal-parietal junction (TPJ)]. These results suggest that avoidant responses to provocation might result from heightened threat anticipation and are associated with reduced perspective taking. Furthermore, our study helps to reconcile conflicting findings on the role of the mentalizing network, the amygdala, and the OFC in aggression.

## Visual Abstract

**Figure:**
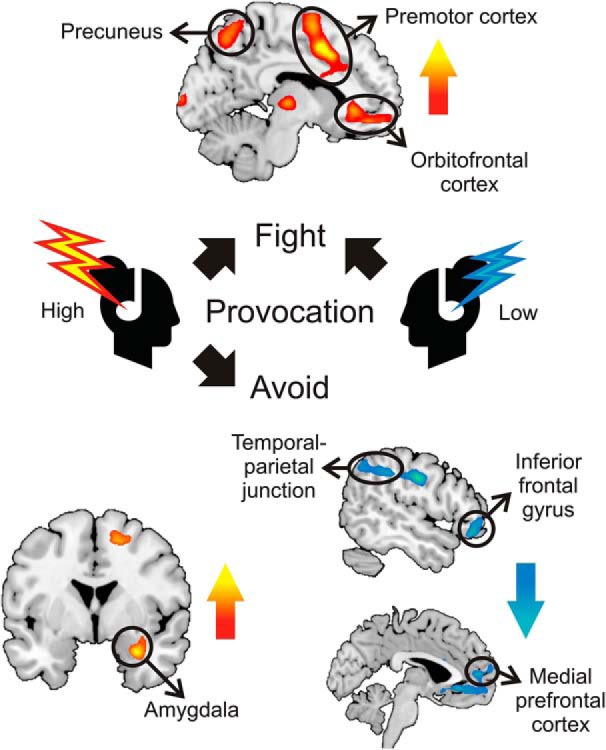

## Significance Statement

Much research has focused on why individuals react aggressively to provocation, but it is also crucial to understand why they avoid confrontation. Here, we investigated the neural basis of aggressive and avoidant responses to interpersonal provocation. Brain regions typically recruited when thinking about others’ intentions were activated when avoiding an encounter, but less so against a highly provoking rival. The basolateral amygdala, a structure involved in rapid threat detection, was more active when participants avoided a highly provoking opponent. This indicates that provocation increases threat anticipation, thereby leading to cognitive and behavioral disengagement. Our study thus identifies plausible neuropsychological processes underlying avoidant and aggressive reactions to provocation and helps to resolve inconsistencies in the neuroscientific literature on aggression.

## Introduction

Human aggression is a complex social behavior with a profound personal and societal impact (Waters et al., 2005). Since many instances of aggression are triggered by perceived provocation (Ander**s**on and Bushman, 2002), many studies have investigated the social and biological factors by which individuals retaliate when provoked (Nelson and Trainor, 2007; Coccaro et al., 2011). However, it is just as pertinent to inquire into why individuals would avoid confrontation, as this could provide cues on how to prevent escalation (Anderson et al., 2008). This point has been hitherto largely overlooked. Investigating both aggressive and avoidant responses to provocation, as well as their neurobiological underpinnings, should thus help to predict the occurrence and development of aggression episodes, and ideally inform preventive and management strategies for aggressive behavior (DeWall et al., 2011).

In laboratory aggression studies, participants are typically exposed to interpersonal threat or provocation (e.g., insults, mild electroshocks) delivered by an ostensible opponent, and experimenters measure to which extent they retaliate. Unfortunately, a nonaggressive option is not always available (Tedeschi and Quigley, 1996). Even when there is one, it usually implies not responding at all, hence not really mimicking a retreat strategy (Ritter and Eslea, 2005). Another line of research focuses on active escape, i.e., instances in which individuals must perform a task to avoid a threat. There are many studies on active escape in both humans (Mobbs et al., 2007; Mobbs et al., 2009; Löw et al., 2015) and rodents (Bravo-Rivera et al., 2015; Ramirez et al., 2015; Campese et al., 2016), but in these cases, threat cues are often nonsocial, subjects cannot retaliate, and avoidance, when possible, is exclusively performance dependent. At best, these tasks mimic encounters with predators, but not reciprocal aggressive interactions with conspecifics (Gross and Canteras, 2012).

In rodents, a circuit formed by the medial amygdala, the ventrolateral subdivision of the ventromedial hypothalamus, and the dorsomedial periaqueductal gray (PAG) is thought to detect danger cues from conspecifics, thereby triggering innate defensive responses (Gross and Canteras, 2012). Indeed, conspecific aggression can be optogenetically controlled by stimulating or silencing these areas (Falkner and Lin, 2014; Miczek et al., 2015; Unger et al., 2015; Falkner et al., 2016). This neural mechanism is roughly conserved in humans, although the medial prefrontal cortex (mPFC) plays an arguably more prominent role therein (Panksepp, 2011; Yu et al., 2014). Specifically, it has been suggested that mPFC encodes social dominance in concert with other brain areas involved in social cognition such as the temporal-parietal junction (TPJ) or the inferior frontal gyrus (IFG) among others (Zink et al., 2008; Bault et al., 2011; Mason et al., 2014). Crucially, Ligneul et al. (2016) have recently shown that victories against a better-performing opponent recruit the mPFC, and that electrically upregulating this region potentiates dominance-based decisions. Taken together, these findings suggest that avoidant and aggressive responses to provocation might partly rely on social-cognitive processes.

In a previous fMRI study, researchers measured fear potentiation (FP) of the startle response as a measure of emotional reactivity to threat, and then set participants to play the Taylor Aggression Paradigm (TAP), an extensively employed competitive reaction time task, against two purported opponents (Beyer et al., 2014b). In line with the results commented in the previous paragraph, participants with higher FP had lower activity in brain areas involved in understanding others such as mPFC, TPJ, precuneus, or IFG (Schurz et al., 2014) when confronting a highly provoking opponent. This pattern of activity in the so-called mentalizing network suggested that individuals high in threat reactivity cognitively disengage from the situation when provoked. This effect was nevertheless unrelated to behavior, presumably because participants did not have the chance to escape and thus overtly manifest their avoidant tendencies.

Here, we developed a version of the TAP that incorporates an avoidance option: the fight-or-escape (FOE) paradigm. Using the FOE, we investigated the neural correlates of aggressive and avoidant decisions against a highly and a lowly provoking opponent. Behaviorally, we expected that participants would be more aggressive against the highly provoking (HP) than the lowly provoking (LP), but would avoid the former more often. Drawing on the studies commented, we had two main hypotheses concerning brain activity. On a within-participant basis, we expected a disengagement of mentalizing regions when avoiding the HP compared with the LP. On a between-participant basis, we hypothesized that lower reactivity to provocation in mentalizing regions, reflecting cognitive disengagement from the aggressive interaction, would be related to escape behavior, and that this effect would be stronger for participants high in trait avoidance.

## Materials and Methods

### Participants

We recruited only female participants to circumvent possible gender differences in competitiveness and approach-avoidance motivation (Kivikangas et al., 2014). We gathered participants through flyers and emails from the local student population. One participant was excluded due to excessive head movements (>3 mm in any direction) and three because they guessed that the paradigm was preprogrammed. Hence, the sample was comprised of 36 participants (mean age = 22, SD = 4) who reported to be free of psychiatric or neurologic disorders. The study was approved by the university Ethics Committee and performed in accordance with the Declaration of Helsinki. Subjects provided informed consent, and were compensated for participation.

### Procedure

On each measurement, we met participants as well as two female confederates. We told them they would play an interactive game with each other, which one of them would play from inside the scanner, and the other two with laptops connected to the scanner. Participant and confederates read the instructions together, and then the participant was taken to the scanner. We placed an MR-compatible pulse oximeter in the thumb of the left hand (see below, Heart rate [HR] data acquisition). After four practice trials, the functional measurement took place (≈30 min). The paradigm was presented through scanner-compatible goggles, with diopter-matched lenses if required. Participants’ responses were recorded with two 4-button devices strapped to their waist. After the TAP, participants fulfilled a series of questionnaires, plus two computerized tasks (see below, Computerized and self-report measures).

### The FOE paradigm

The task was developed as a variant of the TAP (Taylor, 1967). The TAP is a widely used competitive reaction time task that elicits aggression through provocation. Here, we operationalized provocation as an aversive sound blast (i.e., a styrofoam scratching noise). In this version of the TAP, which we called FOE paradigm, participants faced each of the two purported opponents in alternating order, and had the option to avoid a limited number of trials.

**Figure 1.**
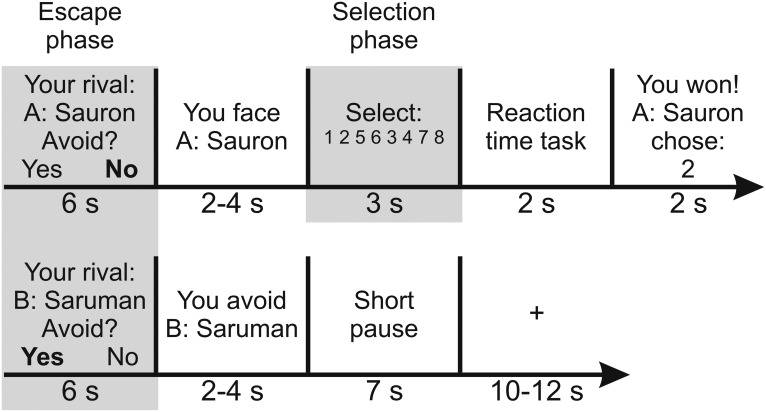
Example trials of the FOE paradigm. Participants confronted a HP and a LP opponent alternatingly. In the escape phase, they could choose whether to fight (upper row) or avoid the encounter (lower row). Fighting led to the punishment selection phase, followed by a reaction time task in which they had to press any button faster than their alleged opponent. In the outcome phase, participants were informed of whether they won or lost and, in the latter case, received the corresponding sound blast through the headphones. If they chose to avoid the trial, they had a short pause. Avoidance decisions were limited to 5 out of 20 trials per run (three runs in total). The fixation cross is only depicted for the avoidance trial, but appeared between all trials regardless of the participant’s decision. For details, see Materials and Methods.

In the escape phase (6 s), participants were informed of which of the two opponents they were playing with and had to choose whether to avoid the encounter. If they did, they waited until the next trial (7 s + inter-trial interval). They could do so a maximum of 5 out of the 20 trials in each of the three runs. They played equally often against each opponent. If they did not avoid, they had to select the loudness of a sound blast (scale 1-8) to be later directed at their purported opponent (selection phase, 3 s). This was followed by the reaction time task (2 s), in which they had to press a button quicker than the rival when a jittered target (0.5 s) appeared. In the outcome phase (2 s), they were informed of whether they had won or lost and of the opponent’s punishment selection. If they lost, they also received the corresponding sound blast. The inter-trial interval (10–12 s) had a randomized variable length (Fig. 1).

The task was preprogrammed such that participants lost two thirds of the trials, equally distributed against both opponents. One opponent selected on average higher punishments (high punisher, HP; range 4–8) than the other (low punisher, LP; range 1–5). The cover story was set in the Lord of the Rings (LOTR) universe (Tolkien, 1954) to engage participants and to make the task easier to understand. Participants were told that they would play as Frodo (protagonist of the novel), and that the opponents would play as Sauron and Saruman (antagonists). The avoidance option was phrased as “putting the Ring on” because said ring confers invisibility to its wearer in the LOTR mythology (Tolkien, 1954). The targets in the reaction time task were three orc pictures from the LOTR movies, presented in random order. For half of the participants the HP was Sauron, and for the other half it was Saruman. The maximal loudness of the sound blast was adapted to each participant’s tolerance. The paradigm was programmed and implemented in Presentation (version 16.5, www.neurobs.com, RRID: SCR_002521).

### Computerized and self-report measures

#### Approach-avoidance task (AAT)

This task was used to measure approach-avoidance motor responses to threat and reward signals, which were operationalized as happy and angry faces, respectively (Roelofs et al., 2009; Volman et al., 2011). Participants played with a joystick (Speedlink Dark Tornado), which they either had to pull or push when seeing a happy or an angry face, depending on the condition. The size of the faces was gradually increased in size when pushing and decreased when pulling, giving the impression that the images approached or receded. Participants should thus be slower to pull angry faces “toward them,” and slower to push happy faces “away,” allowing to calculate an avoidant bias score from reaction times (Roelofs et al., 2005).

Pictures were extracted from the Radboud Faces Database (Langner et al., 2010). We used photographs of 30 persons (15 female), each showing an angry facial expression in one picture, and a happy expression in the other. Pictures of nine different individuals (four female) were used for practice blocks. The pictures were cropped into an oval shape, removing hair, ears and neck.

In a first block, participants had to pull happy faces toward them, and push angry faces away. In a second block, the rule was reversed. The size of the faces was increased or decreased in seven gradual steps. Images had an initial size of 6 cm (≈7.6° of the visual angle), and could be shrunk to 2 cm (≈1.2°) or enlarged to 22 cm (≈13.9°). Each trial started by pressing the joystick’s trigger button, and a central fixation cross of was presented between trials. Each block consisted of 30 happy and 30 angry trials, and we used the same pictures in both blocks. Each block was preceded by a practice run, in which feedback was provided: a green check-mark for correct reactions, and a red cross for errors. The practice run for the first block consisted of 20 trials, whereas the second consisted of 28 trials because participants had to learn the reverse rule. No feedback was provided during the task proper.

The main outcome measure was the response latency until the first movement. We rejected trials in which the first movement was in the wrong direction, as well as trials with responses shorter than 150 ms or longer than twice the participant’s own standard deviation. We calculated an implicit avoidant bias as the pull-push reaction time difference in angry trials, and an implicit approach bias as the pull-push reaction time difference in happy trials (Roelofs et al., 2009). Reaction times per condition were push angry: 573 ± 8 ms (main ± SE), pull angry: 644 ± 16 ms, push happy: 630 ± 15 ms, pull happy: 577 ± 10 ms.

#### Dot-probe paradigm (DPP)

The DPP is a well-established measure of attentional avoidance (MacLeod et al., 1986). In the version employed here, participants were presented with an angry and a neutral face, which were followed by a target (a dot) appearing either on the right or the left side of the screen. In half of the trials, the target was presented in the former position of the angry face (congruent condition) and in the other half in the previous location of the neutral face (incongruent condition). As individuals tend to initially allocate attention to threatening stimuli and then look away (Cooper and Langton, 2006), we programmed the task with a long exposition time (1 s) to measure this general avoidant bias.

Face stimuli were 40 pictures extracted from a set of previously validated videos (Kircher et al., 2013). The pictures were stills of 20 professional actors (nine women) displaying angry and neutral facial expressions. In each trial, two pictures of the same person with a neutral and an angry expression were presented together, to the left and right of the screen center.

Each trial began with a fixation cross being presented for a jittered interval between 500 and 1000 ms. Then, two pictures (4.5 cm/≈3.6° each) of one person with a neutral and angry expression were presented for 1000 ms. At picture offset, a dot appeared to the left or right, at the position where the center of the corresponding picture had been. Participants were instructed to react as quickly as possible to the dot, by pressing the A key on the computer keyboard if the dot was presented on the left, and L if it was presented on the right side.

Participants completed 80 trials in each of the two blocks. Each picture pair was presented four times with the following configurations: angry face and target on the left (congruent); angry face and target on the right (congruent); angry face on the left, target on the right (incongruent); angry face on the right, target on the left (incongruent). Scores were calculated as the difference in reaction time between congruent and incongruent correct trials, such that higher values would indicate attentional avoidance of the threatening stimulus. Reaction times per condition were: congruent: 391 ± 10 ms, and incongruent: 390 ± 11 ms.

Both computerized tasks were run in Presentation (version 17.2, www.neurobs.com) on a Dell Latitude E6400. The monitor had a resolution of 1440 × 900 pixels and a 60 Hz refresh rate. Participants’ head was at a distance of ≈90 cm from the screen during the AAT and ≈70 cm during the DPP (the joystick was removed and the computer was brought closer for the DPP).

#### Questionnaires

Participants fulfilled the harm avoidance (HA) scale from the revised temperament and character inventory (TCI-R) in German (Brändström et al., 2003), a dichotomous 35-item measure with four subscales: anticipatory worry, fear of uncertainty, shyness, and fatigability. We also used the German version of Carver and White’s BIS scale (Strobel et al., 2001), which is based on Gray’s biopsychological theory of personality and is thought to measure punishment sensitivity and general avoidant tendencies. The scale employed here has sevven items, scored on a 1–4 (“completely disagree” to “completely agree”) Likert scale. In addition, participants fulfilled the German version of the Liebowitz Social Anxiety Scale (LSAS; Stangier and Heidenreich, 2005), which uses a 4-point scale to measure fear (“none” to “severe”) and avoidance (“never” to “usually”) of different social situations.

We used a questionnaire to check whether the experimental manipulation had succeeded. Participants rated the unpleasantness of the highest and lowest tones (scale 1–8), and the fairness of their two opponents (1–8). We assessed whether they had been successfully deceived with three qualitative questions (“Have you noticed anything special in the opponents’ behavior?”; “Have you followed any specific strategy during the game?”; and “What do you think this study investigated?”) and in the debriefing. In addition, we administered the 27-item German version of the aggression questionnaire (AQ; Herzberg, 2003), an *ad hoc* translated German version of the revised competitiveness index (RCI; Harris and Houston, 2010) with 14 items, and one extra question inquiring on weekly hours of videogame use. Scores for all questionnaires were: BIS: 19.92 ± 0.60, LSAS-Fear: 15.44 ± 1.65, LSAS-Avoidance: 14 ± 1.72, HA: 12.56 ± 1.01, RCI-Competitiveness: 28.03 ± 1.17, RCI-Contentiousness: 15.25 ± 0.63, and AQ: 2.00 ± 0.06. The internal consistency of all scales was satisfactory (α = [0.780, 0.961]).

### Heart rate (HR) data acquisition

Photoplethysmography was performed with an *in vivo* Precess Model 3160 pulse oximeter attached to the thumb of the left hand. The device had the following technical specifications: saturation precision = 70–100% ± 3%, pulse range = 30–240 bpm ± 3 bpm, pulse accuracy = ±3, LED1 wavelength = 663 nm, LED2 wavelength = 948 nm, LED1 output power = 66.9 uW, LED2 output power = 39.1 uW, pulse duration = 6.06 × 10^−4^ s.

### Neuroimaging data acquisition

We acquired all scans with a 32-channel head coil mounted on a Philips Ingenia 3.0T scanner supporting gradient echo-planar imaging (EPI). We obtained anatomic images with a T1-weighted EPI sequence (180 sagittal slices, TR = 7.7, TE = 3.5, FOV = 240, matrix = 240 × 240 mm, flip angle = 8°, voxel size = 1 mm isotropic). For functional scans, we used a T2*-weighted EPI scanning protocol sensitive to changes in the blood-oxygen-level-dependent (BOLD) signal (47 axial slices per volume, TR = 2.5 s; TE = 25 ms; FOV = 200 mm, matrix = 80 × 80 mm; flip angle = 90°; voxel size = 2.5 mm isotropic). The beginning of each trial of the task (see above, The FOE paradigm) was timed to coincide with the start of a volume to reduce sampling variability in the escape phase, our main epoch of interest. We recorded three consecutive runs of 216 volumes each (i.e., 9 min per run), with five dummy scans at the start of each run to permit steady-state tissue magnetization.

### Behavioral data analysis

We first calculated the cumulative proportion of subjects who used up all avoidance options in each trial, which provides information on participants’ overall strategy (i.e., whether they exhausted all avoidance options early in the run or saved them for later trials). Subsequent inferential statistics were computed with linear mixed-effect models (LMMs, also known as hierarchical or multilevel models), which are appropriate for unbalanced data and permit to model trial-wise behavior (Baayen et al., 2008; Aarts et al., 2015). These analyses were performed with the *lmerTest* package version 2.0–33 (Kuznetsova et al., 2015) implemented in R version 3.1.3. First, we tested the effects of provocation (coded as 0 = low, 1 = high) and run (coded as −1, 0, and 1 for runs one to three) on aggression and avoidance. Provocation and run were defined as fixed-effect factors, whereas subject was defined as a random factor. Additionally, we ran a LMM on trial-wise response latencies in the escape phase with factors decision (coded as 0 = fight, 1 = avoid), run, and provocation, aiming to test whether participants took more time to choose the avoidance or fight options against one or the other opponent. In this model, decision was defined as a participant random-effect variable. We also modeled reaction times in the punishment selection phase and in the reaction time task with factors provocation and run. For avoidance, we fitted a generalized LMM (function *glmer*) with a logit link function for binomial outcomes, whereas all other variables were modeled with standard LMMs (function *lmer*) for continuous outcomes. We report parameter estimates (b) and their associated statistics (*t*/*Z* and *p* values) for all LMMs. Where appropriate, we performed pairwise *post hoc t*/*Z* tests (Tukey-adjusted for multiple comparisons) with the function *lsmeans* (Lenth, 2016). The means/proportions and SEs extracted with *lsmeans* were used to plot the observed effects.

Regarding self-report data, we first analyzed the manipulation check. We inspected whether participants rated the lowest and the highest tone differently, and whether they perceived the high punisher as less fair than the low punisher by means of paired *t* tests. Finally, we tested whether the mean difference in avoidance or aggression between the high and low provoker (i.e., the provocation effect) was related to any of the avoidance or control measures with Pearson correlation coefficients. We only used questionnaires’ total scores, and not subscales, to avoid inflating the number of tests. Only measures related to avoidance or aggression were used to explore further brain-behavior relationships. Significance was set at *p* < 0.05 (uncorrected). These analyses were performed with built-in R functions.

### HR data analysis

We visually inspected the HR data for artifacts, which left 30 participants with complete usable data. We removed linear drifts with the *detrend* function from MATLAB R2015b and computed HR change as the pulse count difference between the 1 s prestimulus baseline and each second of the escape and selection phases (Bradley et al., 2001). Since we expected an initial freezing response to provocation (Lang and Bradley, 2013), we extracted the maximum deceleration (i.e., the minimum value) across the duration of the escape phase for each trial. We then ran a series of LMMs on these scores with within-subject factors decision, run, and provocation as we did for behavioral data. In the selection phase, we expected to observe a preparatory HR acceleration (van Honk et al., 2001), and so we extracted the maximum acceleration in HR relative to baseline. In this case, we fitted a run by provocation LMM.

### Neuroimaging data analysis

We used Statistical Parametric Mapping 12 (SPM 12; Wellcome Department of Imaging Neuroscience, University College London, London, UK, RRID: SCR_007037) implemented in MATLAB R2015b for the analysis of neuroimaging data. We first realigned all scans manually according to the anterior-posterior commissure. We applied standard preprocessing steps, namely slice-timing correction to the middle slice, realignment to the first functional volume, coregistration of anatomic and mean functional images, segmentation of the anatomic image with the standard SPM12 “Segment” function (known as “New Segment” in SPM8), normalization to the native voxel size in Montreal Neurologic Insitute (MNI) space, and smoothing with an 8 mm full width at half maximum (FWHM) Gaussian kernel.

We then fitted two first level models to answer our different research questions. In all cases, regressors in the escape phase were defined to be 6 s long. In a first model, we specified two regressors in the escape phase and two in the selection phase for high and low provocation trials, as well as four 3-s regressors in the outcome phase (won and lost against the high and low provoker). We also modeled the reaction time task (onset of the target), the sound of the punishment in lost trials, and movement parameters derived from realignment as regressors of no interest. The onset of motor responses (i.e., button presses) rather than reaction times were also included as nuisance regressors, since modeling the latter can remove genuine decision-related activity (Grinband et al., 2008). Regressors were convolved with the canonical hemodynamic response function, and we applied a 128-s high-pass filter to remove signal drifts, as well as SPM’s autoregressive function.

To test for differential brain reactivity to the high relative to low provocation, we performed one sample *t* tests contrasting high versus low trials in the escape phase. In addition, we performed multiple regression analyses to probe whether avoidance could predict brain reactivity to provocation across participants. Hence, we regressed brain activity in the high > low contrast in the escape phase against the provocation effect for avoidance (i.e., difference in number of avoided trials between HP and LP). Although our focus was on the escape phase, we also analyzed the selection and outcome phases. In the selection phase, we compared high versus low provocation, and regressed the behavioral provocation effect for aggression (i.e., difference in mean punishment selections between HP and LP) on this contrast. In the outcome phase, we conducted a flexible factorial analysis with factors won versus lost and high versus low provocation, and tested both main effects (won > lost, lost > won, high > low, low > high) and the interaction (won high > won low: lost low > lost high and won low > won high: lost high > lost low) with *t* tests.

In a second set of analyses, we investigated brain activity associated with fight versus avoidance decisions, and whether these could be modulated by provocation. In these analyses, we only included participants who avoided each opponent at least once in the same run (*n* = 27). We did so for two reasons. First, including participants with too few trials can reduce statistical power when conducting ANOVA on unbalanced data (Tibon and Levy, 2015). By including only participants who avoided both opponents in the same run we ensured that a minimum amount of avoidance trials per run (i.e., 2 out of 20 or 10%) was modeled and thereby achieved higher power to detect avoidance-related effects. Such an approach is common practice in fMRI studies conducting performance-dependent contrasts (Rodehacke et al., 2014; Madipakkam et al., 2015; Marchewka et al., 2016). Second, this procedure permits to create a balanced second level design, which ensures orthogonality between the different effects (McFarquhar, 2016). First-level models were fitted identically as before, but included four regressors in the escape phase defined by participants’ decision: avoid high, avoid low, fight high, and fight low. Pauses (7 s) when using the avoidance option were also modeled separately for the HP and the LP. If participants used up all avoidance options in a given run, the escape phase was modeled as a pause, since there was no decision to be taken. If they never decided to avoid or avoided only one opponent in a given run, that run was excluded from the analysis. At the second level we performed a flexible factorial ANOVA with factors decision (avoid vs fight) and provocation (high versus low). Given that there were considerably less avoidance than fight trials, we assumed unequal variance between the levels of this factor. We then tested the main effects of decision (avoid > fight and fight > avoid) and its interaction with provocation (avoid high > avoid low: fight low > fight high and avoid low > avoid high: fight high > fight low).

In all analyses, we applied a whole-brain family-wise error (FWE) corrected threshold of *p* < 0.05 at the cluster level with a cluster-forming threshold of *p* < 0.001 (uncorrected). Anatomic regions were labeled according to the atlases implemented in xjView (http://www.alivelearn.net/xjview8/, RRID: SCR_008642). We extracted mean parameter estimates for each condition within a 7.5-mm radius sphere (i.e., three voxels) around the peak of significant clusters for data visualization using MarsBar (http://marsbar.sourceforge.net/, RRID: SCR_009605).

## Results

### Behavioral and self-report results

**Figure 2.**
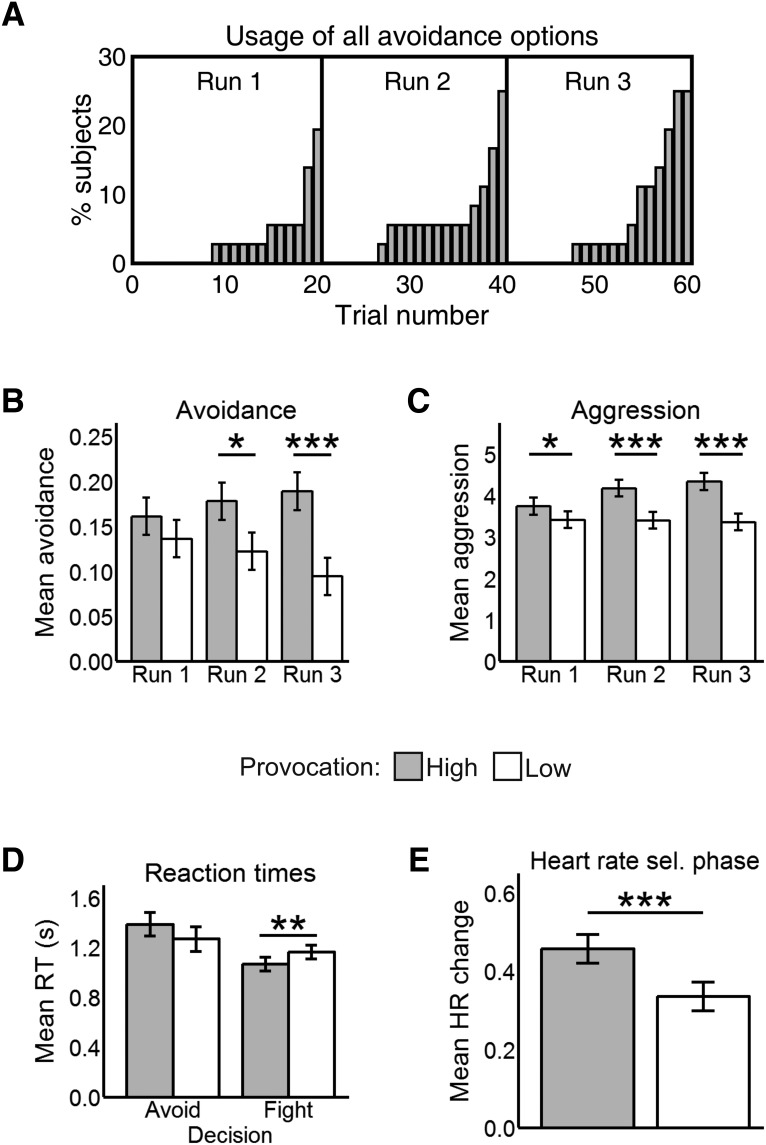
***A***, Cumulative proportion of participants who used all avoidance options in each trial. ***B***, Mean avoidance by run and provocation. ***C***, Mean aggression by run and provocation. ***D***, Reaction times in the escape phase by decision and provocation. ***E***, HR results in the selection phase by provocation. All values in this figure are mean ± SE. *p < .05, **p < .01, ***p < .001.

Participants rated the loudest tone as more distressing than the lowest one (*t*_35_ = 18.03, *p* < 0.001), and perceived the HP to be less fair than the LP (*t*_35_ = 9.20, *p* < 0.001). No >25% of participants (i.e., 9 out of 36) used up all avoidance options in any run. Furthermore, participants tended to exhaust avoidance options toward the end of each run (Fig. 2*A*).

As predicted, participants were more likely to avoid the HP than the LP (b = 0.50, *Z* = 4.00, *p* < 0.001; Fig. 2*B*). In the punishment selection phase, they selected higher punishments against the HP compared with the LP (b = 0.69, *t*_1806_ = 8.86, *p* < 0.001; Fig. 2*C*). There were no main effects of run (both *p* > 0.097). The run by provocation interaction was significant for punishment selections (b = 0.32, *t*_1804.4_ = 3.40, *p* < 0.001) and near-significant for avoidance (b = 0.29, *Z* = 1.91, *p* = 0.055). Hence, we compared avoidance and aggression scores for high versus low provocation in each run to clarify whether participants learned the difference between both opponents over time. For avoidance, the difference between the HP and the LP was significant in the second (*Z* = 2.12, *p* = 0.033) and third runs (*Z* = 3.65, *p* < 0.001), but not in the first (*p* = 0.282). For aggression, it was significant in all runs (run 1: *t*_1802.60_ = 2.40, *p* = 0.016, run 2: *t*_1802.87_ = 5.67, *p* < 0.001; run 3: *t*_1802.60_ = 7.22, *p* < 0.001). The increasing effect sizes indicate that the provocation effect became stronger over time for both avoidance and aggression.

Participants were generally quicker to decide in the escape phase when facing the HP than the LP (b = −0.09, *t*_2091.9_ = −3.34, *p* < 0.001) and over time (b = −0.08, *t*_2085.9_ = −3.55, *p* < 0.001). Importantly, we found a significant interaction (b = 0.21, *t*_2067.20_ = 2.70, *p* = 0.006) between provocation and decision (Fig. 2*D*), such that participants were faster to choose the fight option against the HP than the LP (*t*_2082.95_ = 3.23, *p* = .001), but took a comparable amount of time to avoid each opponent (*p* = 0.112). There were no other main effects or interactions (all *p* > 0.265).

Reaction times in the punishment selection phase were unaffected by run or provocation (*p* > 0.123 for main effect and interaction). In the reaction time task participants became faster over runs (b = −0.02, *t*_1804_ = −2.58, *p* = 0.009), but there were no main or interactive effects of provocation (both *p* > .634).

DPT scores, avoidance scores in the AAT, personality and control measures were not associated with the provocation effect for either avoidance or aggression (all *p* > 0.178). However, participants who rated the HP as more unfair than the LP showed stronger provocation effects for both avoidance (*r* = 0.40, *p* = 0.015) and aggression (*r* = 0.52, *p* = 0.001). Furthermore, participants with a higher approach bias in the AAT (i.e., those who were faster to pull relative to push happy faces) were, at trend level, less avoidant (*r* = −0.32, *p* = 0.054) and more aggressive (*r* = 0.32, *p* = 0.052) when facing the HP relative to the LP.

### Heart rate results

Regarding the escape phase, we found no differences between opponents in heart rate (HR) reactivity (*p* = 0.362), nor a run by provocation interaction (*p* = 0.275). HR did not differ either between fight and avoid decisions (*p* = 0.605), nor was there a decision by provocation (*p* = 0.284) or three-way interaction (*p* = 0.225).

In the selection phase, we found a main effect of provocation (b = 0.12, *t*_1507_ = 4.05, *p* < 0.001; Fig. 2*E*) such that participants had a higher HR increase relative to baseline in HP (0.46 ± 0.03) than in LP trials (0.34 ± 0.03). Time had no main or interactive effects (both *p* > 0.341).

### Neuroimaging results

#### Neural reactivity to provocation in the escape phase

No region was differentially active in the high > low or low > high comparisons in the escape phase. In regression analyses, we did not find any association between brain activity in this contrast and the provocation effect for avoidance across participants.

#### Neural activation in fight versus avoid decisions

**Figure 3.**
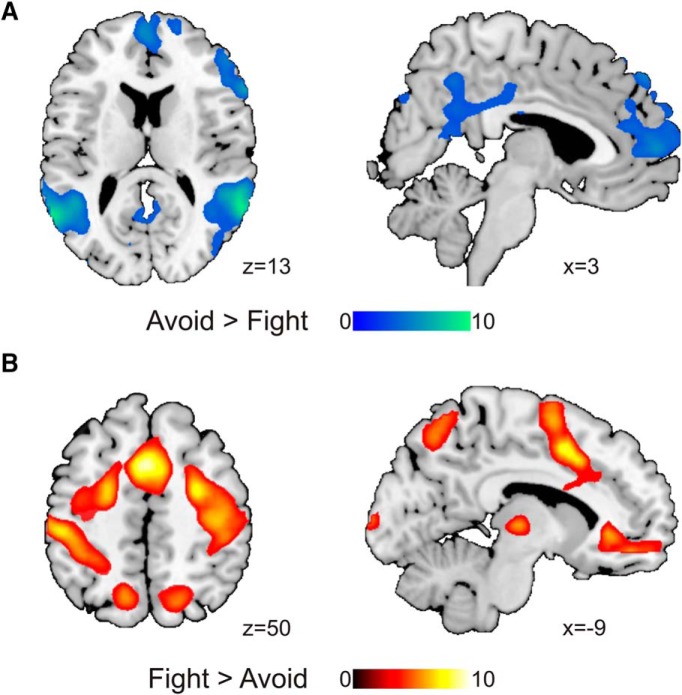
***A***, Avoid > fight contrast. Avoid decisions were linked with activation in regions such as the medial frontal gyrus (mPFC), the TPJ, the PCC extending into the ventral precuneus, and the right IFG. ***B***, Fight > avoid contrast. Fight decisions were associated with increased activation in bilateral somatomotor cortex, OFC, ventral thalamus, and dorsal precuneus. Statistical parametric maps are thresholded and presented at *p* < 0.001, pFWE < 0.05 cluster-level corrected; *N* = 27.

When participants decided to avoid, we observed widespread bilateral activation across the superior temporal sulcus (STS), mPFC, IFG, and posterior cingulate cortex (PCC) extending to the ventral precuneus among other regions (Table 1; Fig. 3*A*). When participants decided to fight, we found increased activation in motor and somatosensory cortex, left orbitofrontal cortex (OFC), dorsal precuneus, ventral thalamus, and middle occipital lobe (Table 1; Fig. 3*B*).

**Table 1.** Brain activity in the escape phase

Region/contrast	k	Peak T	x	y	z
a) Avoid > fight					
Superior temporal gyrus	10182	9.63	65.5	−49.5	20
		9.49	50.5	−42	20
		9.23	58	−52	10
IFG	322	6.43	63	23	12.5
		5.42	55.5	25.5	5
		4.84	50.5	40.5	12.5
Middle frontal gyrus	1067	6.29	25.5	25.5	37.5
Superior frontal gyrus		5.88	18	53	40
Medial frontal gyrus		5.85	0.5	58	7.5
b) Fight > avoid					
Superior frontal gyrus	9856	10.09	−22	−2	65
		9.69	28	−7	57.5
Supplementary motor area		9.60	−7	10.5	45
Anterior cerebellum	193	7.81	30.5	−52	−32.5
		5.55	20.5	−54.5	−22.5
Precuneus	276	7.43	18	−67	55
Ventral thalamus	156	6.06	−9.5	−17	2.5
		4.22	0.5	−29.5	2.5
		3.47	3	−19.5	−5
Superior occipital gyrus	164	5.88	18	−97	15
		5.18	13	−99.5	2.5
c) Avoid high > avoid low: fight low > fight high					
Anterior cerebellum	648	7.33	40.5	−52	−30
Posterior cerebellum		4.55	8	−72	−20
		4.26	18	−72	−22.5
Cuneus	2747	6.67	−17	−57	25
Middle occipital gyrus		6.66	−32	−74.5	25
Cuneus		6.18	13	−79.5	27.5
Posterior cerebellum	287	5.53	−27	−69.5	−27.5
		4.97	−34.5	−52	−30
Lingual gyrus	308	5.45	13	−47	2.5
		4.58	23	−64.5	−5
		3.82	23	−49.5	7.5
Amygdala	182	5.34	30.5	3	−22.5
Temporal pole		5.13	43	10.5	−17.5
Inferior frontal cortex		4.25	30.5	15.5	−22.5
Supplementary motor area	259	4.78	28	10.5	62.5
		4.57	13	−4.5	60
		4.33	13	−14.5	60
d) Avoid low > avoid high: fight high > fight low					
Middle frontal gyrus	4812	7.48	28	53	0
Middle frontal gyrus		6.99	−24.5	40.5	25
Postcentral gyrus		6.30	−32	−22	35
Postcentral gyrus	238	6.72	53	−9.5	35
		5.39	63	−4.5	30
		4.36	55.5	−9.5	22.5
Inferior parietal lobe	232	5.66	55.5	−57	42.5
		5.00	53	−37	40
		4.68	55.5	−64.5	30

*n* = 27, *p* < 0.001, pFWE < 0.05 cluster-level corrected.

#### Interaction between provocation and decision

**Figure 4.**
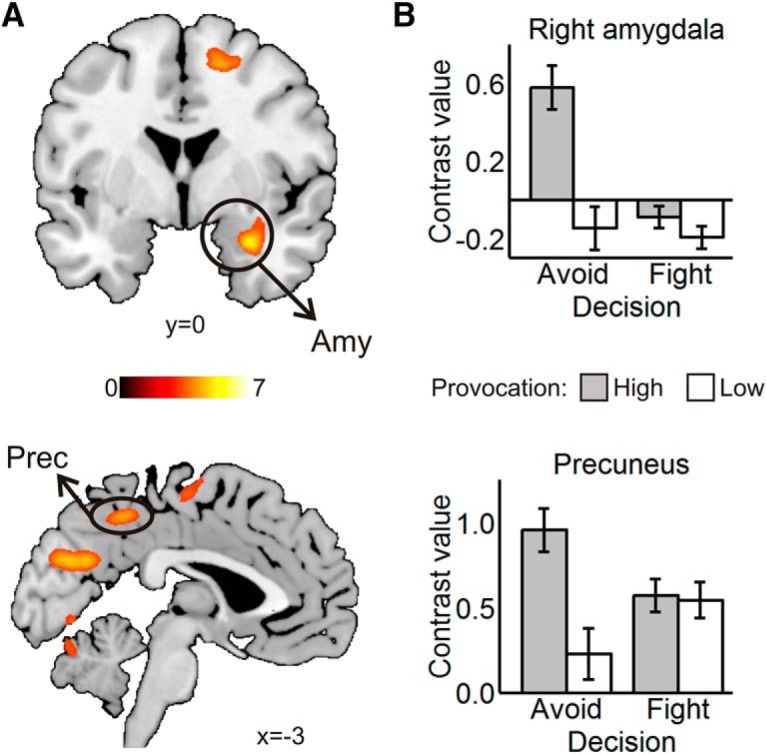
Avoid high > avoid low: fight low > fight high contrast. ***A***, Amygdala (Amy), precuneus (Prec), cuneus, and posterior cerebellar clusters. Statistical parametric maps are thresholded and presented at *p* < 0.001, pFWE < 0.05 cluster-level corrected. ***B***, Contrast values by decision and provocation in basolateral amygdala (above) and precuneus (below). Values are mean ± SE within a 7.5 mm sphere around the local peak; *N* = 27.

The right amygdala, the cuneus extending to the middle precuneus, and bilateral posterior cerebellum showed increased activation when participants chose the avoidance option in the high compared with low provocation condition (Table 1; Fig. 4).

**Figure 5.**
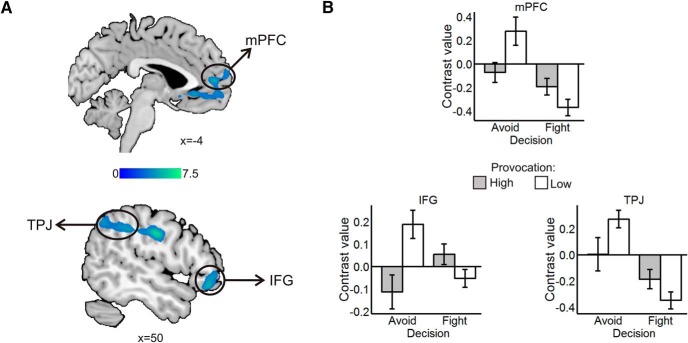
Avoid low > avoid high: fight high > fight low. ***A***, Clusters in sgACC extending into the rostral mPFC, in TPJ, and in IFG. Statistical parametric maps are thresholded and presented at *p* < 0.001, pFWE < 0.05 cluster-level corrected. ***B***, Contrast values by decision and provocation in mPFC (above), IFG (below left), and TPJ (below right). Values are mean ± SE within a 7.5 mm sphere around the local peak; *N* = 27.

The intraparietal sulcus extending to the supramarginal gyrus (SMG), middle frontal gyrus, mPFC, IFG, inferior parietal lobule (IPL) covering the TPJ, and subgenual anterior cingulate cortex (sgACC) showed lower activation when participants avoided the HP relative to the the LP (Table 1; Fig. 5).

#### Neural reactivity to provocation in the selection phase

**Figure 6.**
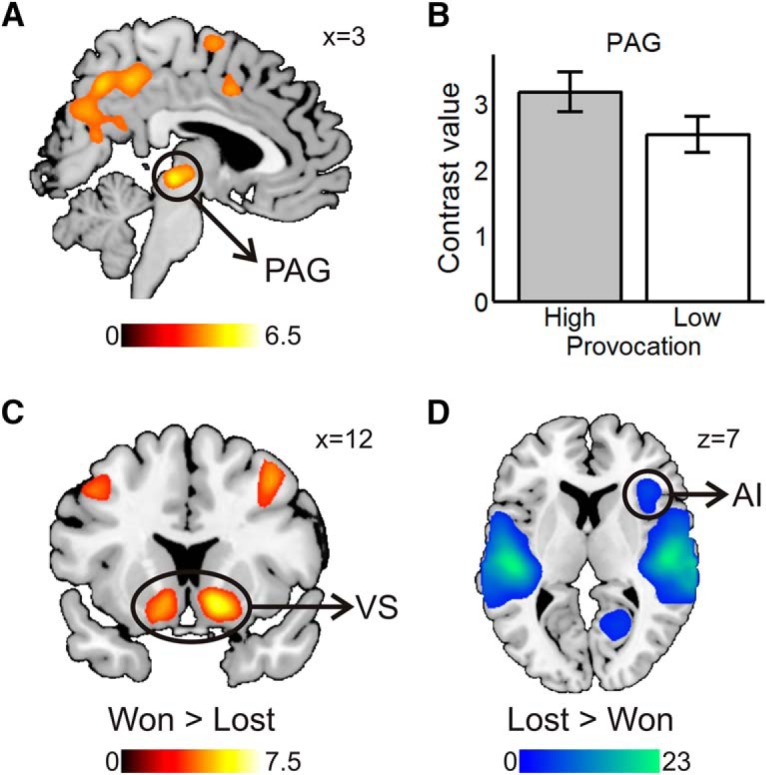
***A***, High > low provocation contrast in the punishment selection phase. High provocation elicited activation in PAG extending to the ventral thalamus, precuneus, supplementary motor area (SMA), TPJ, and IFG among others. ***B***, Contrast values by provocation in the PAG. Values are mean ± SE within a 7.5 mm sphere around the local peak. ***C***, Won > lost contrast in the outcome phase. Winning was associated with activity in VS, middle frontal and inferior occipital areas among others. ***D***, Lost > won contrast in the outcome phase. Losing was related to activity in AI, superior temporal gyrus, and lingual gyrus among other regions. Statistical parametric maps are thresholded and presented at *p* < 0.001, pFWE < 0.05 cluster-level corrected; *N* = 36.

In the punishment selection phase, we found increased reactivity to provocation (high > low) in IFG, IPL covering the TPJ, PCC, precuneus, and the brainstem peaking in the PAG but including the red nucleus and the ventral thalamus (Table 2; Fig. 6*A*,*B*). No clusters survived in the opposite contrast (low > high), and no region was associated with the provocation effect for aggression across participants (Fig. 6).

**Table 2.** Brain activity in the selection and outcome phases

Region	k	Peak T	x	y	z
a) High > low selection phase					
IFG	790	6.50	50.5	20.5	5
		5.48	43	30.5	−2.5
		5.00	28	−4.5	2.5
	429	5.53	43	−52	45
		4.22	55.5	−47	37.5
		4.11	38	−67	55
PCC	1691	5.47	13	−44.5	35
Calcarine sulcus		5.24	−14.5	−62	17.5
Precentral gyrus		4.79	−39.5	−24.5	57.5
PAG	218	5.39	15.5	−24.5	−15
		5.16	3	−24.5	−5
Putamen	129	4.94	25.5	8	−10
		3.68	18	0.5	−15
Supplementary motor area	241	4.90	10.5	−2	67.5
		4.21	8	10.5	62.5
		3.86	−2	−4.5	65
OFC	99	4.88	−32	18	−17.5
		4.21	−32	33	−10
Middle occipital gyrus	93	4.41	−47	−84.5	7.5
Middle temporal gyrus		4.34	−59.5	−69.5	5
		3.91	−52	−74.5	10
Midcingulate cortex	82	4.19	8	5.5	40
		3.73	−9.5	8	40
b) Won > lost outcome phase					
Inferior occipital gyrus	805	7.48	−19.5	−97	−5
		5.60	−27	−94.5	12.5
		5.11	−17	−99.5	12.5
Fusiform gyrus	1568	6.82	35.5	−44.5	−22.5
Inferior occipital gyrus		6.23	28	−92	−5
		5.73	20.5	−97	0
VS	246	6.49	13	13	−10
SMG	724	6.06	−47	−67	35
		4.96	−54.5	−49.5	50
		4.63	−44.5	−57	42.5
Superior temporal gyrus	529	5.67	55.5	−59.5	32.5
		5.06	50.5	−62	45
		3.95	55.5	−47	50
IFG	259	5.29	50.5	38	−10
		4.49	40.5	55.5	−7.5
Middle frontal gyrus	1763	5.04	−29.5	23	45
Medial prefrontal cortex		4.68	−4.5	60.5	15
Middle frontal gyrus		4.54	−14.5	43	42.5
VS	192	4.90	−12	3	−15
		4.68	−12	13	−12.5
		3.33	−9.5	18	2.5
Middle frontal gyrus	537	4.67	38	10.5	47.5
		4.57	23	23	57.5
		4.08	13	35.5	52.5
IFG	463	4.60	−37	50.5	−7.5
		4.60	−47	43	−10
		4.30	−54.5	30.5	2.5
c) Lost > won outcome phase					
Superior temporal gyrus	3055	22.95	−47	−24.5	7.5
		18.72	−54.5	−32	12.5
		17.28	−39.5	−32	12.5
Superior temporal gyrus	3331	22.71	50.5	−19.5	7.5
		19.21	65.5	−27	12.5
AI		6.58	38	25.5	5
Lingual gyrus	293	5.19	20.5	−59.5	5
		3.55	15.5	−72	25

*n* = 36, *p* < 0.001, pFWE < 0.05 cluster-level corrected.

#### Brain activity in the outcome phase

In the outcome phase, won relative to lost trials elicited brain activity in the ventral striatum (VS; Table 2; Fig. 6*C*) as well as in a number of cortical areas, chiefly in the dorsolateral prefrontal and posterior parietal cortex. Lost relative to won trials were associated with large clusters across the superior temporal gyrus, as well as with activation in the lingual gyrus and the anterior insula (AI; Table 2; Fig. 6*D*). There were no main or interactive effects of provocation in the outcome phase.

## Discussion

We investigated the neural correlates of fight and avoidance decisions in response to provocation. Participants avoided the HP more often, but selected louder sound blasts when confronting her. Nevertheless, these behavioral provocation effects were not related to computerized and self-report measures of avoidance on a between-participant basis. Fight decisions yielded increased activation in OFC, dorsal precuneus, and the sensorimotor cortex among other regions. Avoidance decisions were accompanied by increased activity in the mentalizing network, but this effect was less pronounced in high provocation trials. On the other hand, amygdala, precuneus, and posterior cerebellum were more active when avoiding the HP compared with the LP. Our study therefore identifies direct neural correlates of fight-or-flight decisions, and helps to delineate the contribution of OFC, amygdala, and mentalizing regions in aggressive and avoidant responses to provocation.

### Behavioral and cardiac responses to provocation

Participants avoided the HP more frequently and selected higher punishments against her than against the LP. They were also generally quicker to decide when facing the HP, and more so when choosing to fight her. In the selection phase and reaction time task, subjects became quicker over time independently of the opponent or the decision, implying that they learned the task dynamics. Importantly, participants did not generally use all avoidance options. When they did, it was in the last trials, when the end of the run neared. This suggests that participants might have also pondered whether to use up avoidance options or save them for later, which, as pointed out by an anonymous reviewer, adds another layer of cognitive complexity to the decision process. We did not find the expected deceleration in HR in the escape phase, likely because having the opportunity to escape conferred participants a sense of safety (Lang and Bradley, 2013). In the selection phase, however, HR increased when facing the HP, reflecting a typical circa-strike physiologic response to an incoming threat (Lang and Bradley, 2013; Haller et al., 2014). The magnitude of this response was small due to the short duration of this epoch (i.e., 3 s). Overall, results suggest that the FOE paradigm was able to successfully provoke participants and motivated genuine fight and flight responses.

There was no direct relationship between any measure of trait avoidance and avoidant behavior in the FOE. Rather, avoidance and aggression were related to fairness ratings, indicating that participants’ behavior depended more on transient appraisals of the opponent than on broad avoidant tendencies. Alternatively, variability in avoidance might have been too low to detect correlations with personality (Bates et al., 1996). It can also be that the computerized avoidance tasks inadequately captured the construct of interest, given that the DPT has shown poor reliability in nonclinical samples (Schmukle, 2005). Notably, the AAT approach bias toward happy faces was marginally associated with decreased avoidance and increased aggression against the HP relative to the LP. This indicates that participants with high approach motivation were more sensitive to provocation, and is in line with theories suggesting than anger and aggression are approach-driven behaviors (Carver and Harmon-Jones, 2009; Berkowitz, 2012). The finding that participants were faster to select the fight, but not the avoidance, option against the HP also fits this notion. Further developments of the FOE could include, e.g., a shorter time limit to decide and/or additional tradeoffs for fight and avoid decisions. Such constraints would better recreate real world aggression episodes, in which responses to provocation are likely adopted after one-shot, impulsive decisions (Lowe and May, 2011; Simons et al., 2015).

### Neural responses to provocation

We found no general differences in neural activation between the HP and the LP in the escape phase despite the stark contrast in behavior toward each opponent. In previous studies, differential brain reactivity to provocation was only observed when deciding the punishment intensity (Krämer et al., 2007; Beyer et al., 2014b), which presumably entails qualitatively different cognitive processes than choosing whether to avoid or fight. The latter decision might have been complex enough to constrict differences in neural reactivity to provocation, which were only visible within avoid and fight trials. This is supported by the widespread brain activity observed in the avoid versus fight contrasts, and the greater reaction times in avoid relative to fight decisions.

When selecting the punishment, some areas of the mentalizing network (IFG, TPJ, precuneus) were activated in high relative to low provocation trials. This could reflect a more intense deliberation of the consequences of punishing the HP, as is often assumed in competitive or bargaining paradigms (Krach et al., 2008; Assaf et al., 2009; Polosan et al., 2011). This effect was accompanied by increased activity in the PAG extending to the ventral thalamus and other midbrain nuclei, an established defensive reaction to imminent threat (Mobbs et al., 2007; Mobbs et al., 2009). Remarkably, no mPFC activity was observed in this epoch. This finding is in agreement with studies reporting a shift from prefrontal to subcortical activation as a function of threat proximity (Mobbs et al., 2007; Mobbs et al., 2009). Across studies, the mPFC is less strongly recruited in tasks involving quick, one-shot judgements, than in tasks that require inferring stable characteristics (Schurz et al., 2014). Hence, provocation seems to foster alertness and rapid social-cognitive processing in the seconds preceding the aggressive encounter. It is noticeable that other studies with the TAP did not report PAG reactivity to provocation during punishment selection (Krämer et al., 2007; Beyer et al., 2014b). This might be due to the fact that the punishment selection phase here was shorter (3 vs 6 s), so the sense of incoming threat was probably heightened.

### Neural activation underlying fight decisions

When participants decided to fight, we observed increased activity in OFC, sensorimotor regions, the ventral thalamus, and the bilateral precuneus. Somatomotor activation in retaliatory decisions probably reflected lower-level preparatory processes, which might have been exacerbated by the potential value of the task’s outcomes (Iyer et al., 2010). Importantly, this motor activity is unlikely due to differences in reaction times between fight and avoid decisions, which were small in absolute value (around 115 ms) and had been controlled for by including button presses as a nuisance regressor. Medial OFC activation was also increased when participants decided to fight relative to avoid. This shows that the function of the OFC in aggression is not confined to impulse control (Blair, 2001; Mehta and Beer, 2009). Instead, our results indicate that this area accomplishes a more general evaluative role (Stalnaker et al., 2015). From this perspective, the observed OFC activation could correspond to threat assessment (Beyer et al., 2015), and/or vindictive approach motivation (Seymour et al., 2007). Notably, the ventral thalamus was recruited in fight relative to avoid decisions. This subregion has been proposed to integrate motivational and motor proprioceptive inputs, thereby contributing to action selection (Bosch-Bouju et al., 2013). The above commented somatomotor activity and the fact that the PAG cluster observed in the selection phase extended into the ventral thalamus concur with this interpretation.

### Neural activation underlying avoid decisions

In a previously commented fMRI study, participants with high emotional reactivity to threat had less mentalizing network activity when facing a provoking opponent, but there was no relationship between this effect and aggressive behavior (Beyer et al., 2014b). Here, by giving participants an escape option, we expected to observe a direct link between threat-induced deactivation of mentalizing regions and active avoidance. Indeed, activity in mentalizing and “mirror neuron” regions was generally increased when deciding to avoid an aggressive encounter, but was relatively reduced when avoiding the HP. This suggests that participants engaged in mentalizing processes during avoidance decisions, but disengaged from the situation when they perceived high threat, i.e., in HP trials. This is consistent with the reduced reaction times for these trials, with the increased amygdala activation, and with studies showing that social stress disrupts social cognition both at the behavioral (Smeets et al., 2009) and neural level (Nolte et al., 2013). Nonetheless, our interpretation is partly based on reverse inference (Hutzler, 2014), and so alternative explanations, such as, e.g., reduced cognitive effort (Halko et al., 2009) could also account for these effects. In fact, considering that the escape phase was relatively long (i.e., 6 s), we might have captured not only decision-related but also postdecision cognitive processes. This might especially concern activation in the rostral prefrontal cortex and the inferior parietal lobe, which increases during post-decision evaluation and correlates with self-reported uncertainty about the chosen option (Wan et al., 2016). Provocation might thus impair the cognitive processes leading to avoidance decisions as well as the reevaluation of such decisions. On the other hand, mPFC and IFG activity in avoidance decisions could also correspond to the experience of safety, given that relief from pain has been associated with increased activation in these areas (Leknes et al., 2011). If that was the case, the sense of relief and the corresponding BOLD signal in these regions should be greater when avoiding the HP, but we observed the opposite pattern. Relief is therefore unlikely to explain the present results. All in all, we deem it reasonable to assume that activity in these areas corresponds at least in part to social-cognitive processes, although their exact nature and timing (i.e., pre- vs postdecision) cannot be ultimately clarified with the present data.

Remarkably, regions typically regarded as part the mirror neuron system such as the anterior SMG showed a similar pattern of activity in avoid decisions as mentalizing ones. Mirror neurons are thought to be involved in automatic action perception, whereas mentalizing regions contribute to the more complex understanding of others’ cognitive and emotional state (Yang et al., 2015). Although some authors argue that these processes are independent (Catmur, 2015), others construe mirror activity as necessary for higher-level mentalizing inferences (Tidoni and Candidi, 2016). Our data suggests that both systems have a convergent role in deciding how individuals respond to provocation. Supporting this formulation, mirror and mentalizing systems increase their coupling during real-time social interactions (Sperduti et al., 2014), and reactivity to emotional stimuli in the precentral gyrus, a motor mirror region, has been related to aggression (Beyer et al., 2014a).

Unlike the rest of the mentalizing network, we observed a spatial gradient in the precuneus. Dorsal regions were recruited in fight responses, ventral parts were involved in avoid decisions, and the middle area was specifically activated when avoiding and when about to face the HP relative to the LP. Our data agrees with the proposed functional segregation between a sensorimotor and a limbic precuneus (Margulies et al., 2009), and suggests that the differential activation of these subareas might contribute to either aggressive or avoidant responses to provocation.

We found that the right basolateral amygdala was more active when avoiding a highly relative to a lowly provoking opponent. Amygdala reactivity to threat has been linked to aggression (Lotze et al., 2007; Gospic et al., 2011; McCloskey et al., 2016). Albeit this is often interpreted as an approach-driven phenomenon (Beaver et al., 2008), the paradigms used in these studies lack an avoidance option. The present data implies that the amygdala is not involved in either approach or avoidance per se (Fernando et al., 2013; Weymar and Schwabe, 2016), but potentiates defensive behavior adaptively. If escape is possible, amygdala activation will favor avoidance, if not, it will facilitate aggression (LeDoux, 2003; Lang and Bradley, 2013). Furthermore, our results indicate that this structure can signal threat proactively, thereby contributing to controlled decision processes (Pessoa, 2010).

It is also worth noting that posterior cerebellar activity closely resembled that of the amygdala, rather than that of the motor cortex. Activation in the cerebellum was highest when avoiding the HP, and its peak was located on its posterior aspect extending to the vermis. The latter subregion is often termed *limbic* cerebellum (Schmahmann et al., 2007), and is thought to be involved in inferential social-cognitive processes (Van Overwalle et al., 2014). However, since the function of the cerebellum in higher-order cognition is still far from clear (Koziol et al., 2014), its precise contribution to avoidant behavior cannot be readily delimited.

In summary, participants showed increased amygdala activity, reduced mentalizing network activity, and short reaction times in trials in which they avoided the HP. The most plausible explanation of these results is that, on a trial-wise basis, perceived threat caused participants to avoid the opponent both cognitively (not thinking about her intentions) and behaviorally (choosing not to engage in the confrontation). This is consistent with the proposed role of the amygdala in coordinating cortical responses to threat (Pessoa and Adolphs, 2010).

### Brain activity in the outcome phase

Winning relative to losing was linked to activity in the VS. This midbrain dopaminergic structure is thought to code for both general and social rewards (Sescousse et al., 2013). Indeed, VS activation after wins is a highly robust finding in fMRI studies with the TAP (Krämer et al., 2007; Brunnlieb et al., 2013; Beyer et al., 2014b; Emmerling et al., 2016) as well as in other competitive paradigms (Delgado et al., 2008; Bault et al., 2011; Kätsyri et al., 2013). Losing was linked to large clusters of activity in the superior temporal gyrus, peaking in the auditory cortex and hence attributable to the sound blast. However, we also observed a defeat-related cluster on the AI, again mimicking previous findings (Krämer et al., 2007; Beyer et al., 2014b) and presumably corresponding to the obnoxiousness of the punishment (Lamm et al., 2011). This might also account for lingual gyrus activity, which has been related to aversive learning with auditory stimuli (Miskovic and Keil, 2014; McTeague et al., 2015; Gu et al., 2016). Results in the outcome phase thus replicated established effects, arguing for the reliability of the paradigm.

### Limitations

Some shortcomings of the study should be kept in mind. First, the limited temporal resolution of fMRI does not permit to reliably isolate brain activity preceding the decisions, which were taken on average in little >1 s. This also implies, as commented earlier, that brain activity in the escape phase might partly reflect postdecision cognitive processes. Electroencephalography could better track the temporal dynamics of such a quick process (Gluth et al., 2013). Note also that onsets and data acquisition in the escape phase were synchronous, which might compromise statistical power. We did so because the crucial contrasts in this study involved a reduced number of trials, so that making onsets and acquisition asynchronous could add unwanted variability. Furthermore, at relatively short sampling rates (i.e., below 2.68 s), both methods yield similar estimates of the BOLD response (Miezin et al., 2000). Second, as our sample was limited to healthy young women, it should be tested whether similar results can be obtained in other populations. Third, we used HR to keep comparability with previous studies (van Honk et al., 2001; Lang and Bradley, 2013), but the relationship between vagal input and HR is not linear and might be better captured by heart period (Berntson et al., 1995). Fourth, our paradigm did not tap important variables that can influence how one responds to provocation, such as presence of bystanders (Vasquez et al., 2013). This is an interesting venue for future studies with the FOE.

## Conclusions

We explored aggressive and avoidant reactions to interpersonal provocation, as well as their underlying neurophysiological signature. Cardiac and behavioral data suggest that the FOE, our newly developed competitive task, successfully provoked participants. We showed that avoidance was related to activity in mentalizing regions, whereas retaliation was associated with OFC and somatomotor activation. Activity in some areas was however modulated by provocation. Specifically, the right amygdala was upregulated when avoiding a provoking opponent, whereas certain mentalizing and mirror regions (mPFC, TPJ, IFG, SMG) showed relatively decreased activation. Taken together, our results indicate that avoidant responses to provocation might stem from anticipatory threat signaling and are associated with reduced perspective taking. Moreover, our study suggests that threat escapability is a major situational factor that should be considered in laboratory measures of aggression.

## Synthesis

Reviewing Editor: Daniela Schiller, Mount Sinai School of Medicine Decisions are customarily a result of the Reviewing Editor and the peer reviewers coming together and discussing their recommendations until a consensus is reached. When revisions are invited, a fact-based synthesis statement explaining their decision and outlining what is needed to prepare a revision will be listed below.

The authors have responded to most of the concerns, but a few issues remain:

• Series of 1st level fMRI models instead of one combined model. Authors replied: “We must use different models when comparing avoidance vs fight decisions because some participants did not use the avoidance option at all, or only against one opponent, and one cannot define a regressor without any events. In model 1, we could include all participants because we were only testing for differences between high and low provocation, regardless of participants' decisions.”

This argument is not compelling. The authors can use a combined first level model and in those participants that lack one type of decision, leave out the regressor pertaining to that decision. They can then do exactly the same kind of second level test as they are doing now. This combined model will be more accurate: currently some task-related brain activation is unmodelled in most participants, and given the event-related design, these unmodelled responses can spuriosly be explained by a regressor relating to another task condition.

• Use of LMMs: it was unclear whether choice data was analysed with a generalised LMM (glmer in R) with logit link function as it should, or a standard LME with identity link function?

• Heart rate vs. heart period: the authors should cite the relevant reference suggesting that heart rate does not scale linearly with the autonomic input (which presumably scales with the psychological process under study) and mention this as a limitation.
